# Supplementary material for: Pooled prevalence and associated factors of health facility delivery in East Africa: Mixed-effect logistic regression analysis
Source: PLoS One. 2021 Apr 23;16(4):e0250447. doi: 10.1371/journal.pone.0250447 (PMC8064605; doi:10.1371/journal.pone.0250447)
Supplement: Attachment — Submitted filename: review comments_healthfacility_PLosONE.docx [file pone.0250447.s001.docx]

I would like to thank the authors for their submission on this important topic. In summary, this manuscript requires assistance from a native English speaker to improve the grammar and sentence structure, as well as the organization of the material. I found these issues to be rather distracting while reading the manuscript. Secondly, I have made suggestions for improving different sections of the manuscript below. I have also included some suggested rewrites for some statements in the manuscript.

*Introduction*

The authors did not make a clear case for the importance of a pooled study. Decision-making is typical at national and sub-national levels in individual countries so it is not clear what a pooled prevalence study adds. How would a study like this influence policy within each country? The question of “why is this important” is not addressed.

*Methods*
Data Source: Please provide a citation for your data sources, specifically, the DHS program

Please provide a description of your dataset. How many women responded to each question by country? How many responded “Yes” or “No”? How much missing data did you have? What was the DHS year for each country’s dataset? How did you decide on the independent variables?

Please conduct sensitivity analysis to investigate the impact of having more participants from Kenya in this. How does this influence the study findings?

*Discussion*

This section needs to be better organized. The discussion should be used to summarize the study findings, discuss its implications and make recommendations. What does the study findings imply for progress towards the 2030 SDGs? What can be done to improve health facility delivery in East Africa based on the study findings? While it is important to highlight how the study findings is related to existing literature, it is important that actionable insights are drawn from the study

Lines 212 – 214: It seems the aims of the study as stated here differs from what was declared in the introduction

*Suggestions for improving grammar*

Lines 54 – 56: Maternal mortality has decreased significantly in developed countries (3) but SSA continued to account for 66% of maternal deaths worldwide

Suggestion: “continued” should be continues

Lines 58 – 59: As key strategies for reducing maternal and infant mortality, the World Health Organization (WHO) recommended a health facility delivery

Suggested rewrite: The World Health Organization recommends health facility delivery as a key strategy for reducing maternal and infant mortality

Lines 63 – 64: The proportion of institutional delivery serves as a measure of progress towards maternal and infant mortality reduction

Suggested rewrite: The proportion of institutional delivery serves as a measure of progress towards reductions in maternal and infant mortality.

Lines 64 – 67: The East African countries that include Burundi, Ethiopia, Comoros, Uganda, Rwanda, Tanzania, Mozambique, Madagascar, Zimbabwe, Kenya, Zambia, and Malawi are among the world's poor countries with accessibility and affordability of maternal health care services

Suggested rewrite: Globally, East African countries rank the lowest for affordability and accessibility of maternal health services, and have the greatest share of maternal deaths.

Line 175: “was” should be “were”
