# Supplementary material for: Pooled prevalence and associated factors of health facility delivery in East Africa: Mixed-effect logistic regression analysis
Source: PLoS One. 2021 Apr 23;16(4):e0250447. doi: 10.1371/journal.pone.0250447 (PMC8064605; doi:10.1371/journal.pone.0250447)
Supplement: Attachment — Submitted filename: Point by point response.docx [file pone.0250447.s002.docx]

**Point by point response**

**Manuscript Title**: Pooled prevalence and determinants of health facility delivery in East Africa: A pooled analysis of Demographic and Health Surveys/ Pooled prevalence and associated factors of health facility delivery in East Africa: A pooled analysis of Demographic and Health Surveys

**Manuscript ID**: PONE-D-20-19261

**Dear editor/reviewers**:

Thank you for giving us the chance to revise the manuscript. The comments are too imperative, which are important for improving the quality of our paper. We have addressed all of the concerns raised and these modifications are also incorporated in the revised manuscript.

**Response to reviewers’ comment**

1. I would like to thank the authors for their submission on this important topic. In summary, this manuscript requires assistance from a native English speaker to improve the grammar and sentence structure, as well as the organization of the material. I found these issues to be rather distracting while reading the manuscript. Secondly, I have made suggestions for improving different sections of the manuscript below. I have also included some suggested rewrites for some statements in the manuscript.

**Authors’ response**: Thank you for the comments. We extensively modified the sentence structure and any typographical errors with the help of experts. Besides, we consider your suggestions as well as your constructive comments and we modified the manuscript. (See the revised manuscript)

2. Introduction, the authors did not make a clear case for the importance of a pooled study. Decision-making is typical at national and sub-national levels in individual countries so it is not clear what a pooled prevalence study adds. How would a study like this influence policy within each country? The question of “why is this important” is not addressed.

**Authors’ response**: Thank you reviewer for the comments. As you stated very well decision-making is typical at the national and sub-national level in each country, considering this issue we have reported the prevalence of health facility delivery for each country as well we have reported the prevalence of health facility delivery at the East Africa level to know what health facility level looks like as compared to other regions. Just the main thing we use pooled analysis is not only to know the pooled prevalence but also to increase the statistical power of the study to detect the true effect of independent variables. As you know when we pool the DHSs of the 12 East African countries, the sample size increases and could result in the corresponding improvement in the power of the study and we would get valid evidence. Besides, the DHS of the 12 East African countries uses similar study design and sampling procedures, and therefore the estimate we got in this study is reliable to use for policymakers. (See the background and Discussion section, line 98-101, and line 246-250, page 5 and 11)

3. Methods, data Source: Please provide a citation for your data sources, specifically, the DHS program

**Authors’ response**: Thank you reviewer for the comments. We cited the DHS program address. (See the revised manuscript, line 107, and page 5)

4. Please provide a description of your dataset. How many women responded to each question by country? How many responded “Yes” or “No”? How much missing data did you have? What was the DHS year for each country’s dataset? How did you decide on the independent variables?

**Authors’ response**: Thank you reviewer for the comments. There was no missing as we kept our study participants as women who gave birth within five years preceding the survey and present the study sample in each country through the table. We retrieved the independent variables from the DHSs based on literature. Besides, we select variables for the final model using the LASSO and Elastic Net method in addition to the bivariable analysis. (See Table 1)

5. Please conduct a sensitivity analysis to investigate the impact of having more participants from Kenya on this. How does this influence the study findings?

**Authors’ response**: Thank you reviewer for the comments. We reported the prevalence using forest plots but the study was not a meta-analysis. This study was a pooled data analysis, just to know the prevalence of health facility delivery and associated factors in East Africa and in each country based on the DHSs data that have the same design as well as sampling procedure. So, our aim is not to get the pooled estimate like in metanalysis and the I-squared reported in this is not to show the heterogeneity. Here in this study, the unit of analysis was individuals but not studies like a meta-analysis. Overall, our study aimed to use to pooled DHS data to increase the statistical power of the study to detect the true effect of the variables. Besides, we aimed to assess whether health facility delivery utilization has been varied across countries in East Africa, and if we remove Kenya, we can not answer it. Furthermore, we have checked whether the estimates varied with the presence and absence of Kenya, and the result showed there was a difference in the prevalence of health facility delivery but not in the OR.

6. Discussion, this section needs to be better organized. The discussion should be used to summarize the study findings, discuss its implications and make recommendations. What does the study findings imply for progress towards the 2030 SDGs? What can be done to improve health facility delivery in East Africa based on the study findings? While it is important to highlight how the study findings is related to existing literature, it is important that actionable insights are drawn from the study, Lines 212 – 214: It seems the aims of the study as stated here differs from what was declared in the introduction.

**Authors’ response**: Thank you reviewer for the comments. We summarize the study findings and their implications in the Discussion section of the manuscript. Besides, we compare our findings with previous studies and we wrote out the possible explanations. (See the revised manuscript)

7. Suggestions for improving grammar

- Lines 54 – 56: Maternal mortality has decreased significantly in developed countries (3) but SSA continued to account for 66% of maternal deaths worldwide

Suggestion: “continued” should be continues

**Authors’ response**: Thank you reviewer we modified it. (See the Background section, line 67, page4)

- Lines 58 – 59: As key strategies for reducing maternal and infant mortality, the World Health Organization (WHO) recommended a health facility delivery

Suggested rewrite: The World Health Organization recommends health facility delivery as a key strategy for reducing maternal and infant mortality

**Authors’ response**: Thank you reviewer for the comments. We rewrite it. (See the background section, line 70-71, page 4)

- Lines 63 – 64: The proportion of institutional delivery serves as a measure of progress towards maternal and infant mortality reduction

Suggested rewrite: The proportion of institutional delivery serves as a measure of progress towards reductions in maternal and infant mortality.

**Authors’ response**: Thank you reviewer for the comments. We rewrite it. (See the Background section, line 71-72, page 4)

- Lines 64 – 67: The East African countries that include Burundi, Ethiopia, Comoros, Uganda, Rwanda, Tanzania, Mozambique, Madagascar, Zimbabwe, Kenya, Zambia, and Malawi are among the world's poor countries with accessibility and affordability of maternal health care services

Suggested rewrite: Globally, East African countries rank the lowest for affordability and accessibility of maternal health services, and have the greatest share of maternal deaths.

**Authors’ response**: Thank you reviewer for your suggestion. We rewrite it. (See Background section, line 77-79, page 4)

- Line 175: “was” should be “were”

**Authors’ response**: Thank you reviewer for the suggestion. We rewrite it. (see the discussion section, line 263, page 12)

**Response to Reveiwer#2**

The authors make an important contribution to our understanding of the factors associated with health facility delivery. The main advantages of this paper are 1) it uses pooled DHS data from multiple countries, which is a new approach, and 2) the methodological approach is robust and appropriate. The paper is very well organized and written is terms of structure, flow of ideas and coherence. However, there are numerous language errors and awkward sentences so the paper could benefit from professional editing.

**Authors’ response**: Thank you, reviewer. We extensively modified the body of the manuscript for any language error with the help of language experts at the University of Gondar. (See the revised manuscript)

1. I think pooling DHS data is an important contribution of this paper. However, the authors do not say this approach is useful or worth pursing in the paper. The authors should explicitly and clearly explain this.

**Authors’ response**: Thank you reviewer for your comments to strengthen our paper. We used pooled data analysis for this study. As you stated, pooling DHS data is very important to increase the statistical power of the study to detect the true effect of the independent variables in addition to providing the prevalence of health facility delivery at the East Africa level as well as in each country. This is important for WHO as well for international programs to design targeted interventions. Besides, we used the advanced statistical analysis technique that was multilevel analysis, this could be helpful to provide valid evidence for policymakers and program planners. We stated in the background and discussion section in a short and precise manner. (See the background and Discussion section, line 98-101, and line 246-250, page 5 and 11)

2. It is not clear what are the years for these datasets? Are you all for the same year or difference years? And what did the authors do to make sure that the time dimension is not influencing the results?

**Authors’ response**: Thank you reviewer for the comments. The years for these datasets were from 2008/09 to 2018, and to detect whether the time dimension was influencing the study results by categorizing years into two as 0 “If the study period ≤2015” and 1 “if the study period was > 2015” the categorization was based on the millennium development goal as during MDG and during SDG. But unfortunately, it was not eligible for the final model as it has a p-value greater than 0.2 in the bi-variable analysis. Besides, we have done a chi-square test whether the year of the survey was significantly associated with health facility delivery but it was not significant. (See Table 1)

3. There are issues with merging DHS datasets from different countries related to the use of DHS instrument itself in each country and data consistency issues that must be at least covered in the limitations section.

**Authors’ response**: Thank you reviewer for the comments. The DHS uses similar measurements for the variables in each country and the data are consistent. We have checked the consistency of measurement of variables before we do the analysis and the variables were consistently measured. Besides, the variables we have used in this study may not need that much-sophisticated measurement as it was self-report from the respondents through interviews. As a limitation, we have stated the issues related to the study design used in DHS and the variables missed in DHS but that was important for this study.

4. Some of the statements are missing a source/reference. For example, on Page 10, line 215, the reference for the mentioned study is not listed.

**Authors response**: Thank you reviewer for the comments. We provide an appropriate citation for the statements we have used in the manuscript. (See the revised manuscript)

5. Page 11, line 246, “The potential reason could be that educated husbands can include women making decisions about the use of maternal health services in health care.” The way this is written implies that decisions about women’s health are naturally the responsibility of men but educated men include women in the decision. I do not think this is what the authors intended to say so they should write this point more carefully.

**Authors’ response**: Thank you reviewer for the comments. We rewrite it. (see the Discussion section, line 283-289, page 13)

6. I found that the weakest part of the paper is the discussion and more so the conclusions. The discussion should go beyond simply saying when the results agree and disagree with other studies

**Authors’ response**: Thank you reviewer for the comments. We rewrite it extensively. (See the revised manuscript)

7. The study finds that "country, urban residence, maternal education, husband education, multiple gestations, wanted pregnancy, ANC visit during pregnancy, middle and rich wealth status, having an occupation, and married marital status was positively associated with health facility delivery." These results are hardly surprising; this is what we know already. The study only confirms what we already know. The authors should highlight how they believe this study adds value and contributes to a better understanding.

**Authors’ response**: Thank you reviewer for the comments. We had stated in the discussion and conclusion section of the study. This study adds both statistical and public health value. Regarding statistical value, this study was done by pooling DHSs data conducted in 12 East African countries and this increases the statistical power of the study to detect the true effect size. Besides, we applied the mixed-effect analysis as since DHS data has hierarchical nature, therefore, the findings are reliable as it was analyzed using the advanced model. Regarding the public health aspect, this study was conducted at the East Africa level and we get the pooled estimate and the pooled estimate of health facility delivery in East Africa and the prevalence of health facility delivery for each country as well as factors associated with it. These findings are reliable as it was based on weighted large data using the advanced model and this could help to design evidence-based international and national public health programs.

8. The conclusions are quite vague and not helpful. The authors should discuss policy implications of their work. “Therefore, the governmental and non-governmental organizations should scale up their programs to encourage women education and ANC service utilization for pregnant women.” This statement is completely generic and unhelpful. Why should non-governmental organizations listen? An adequate well-written policy implication section should be included in the conclusions section.

**Authors’ response**: Thank you reviewer for the comments. We rewrite it. (See the conclusion section, line 360-374, page 16)

9. Language

• Page 2, line 17 – “But still….” Is an informal way to express this idea

• Page 3, line 44- “ the government and non-governmental…. what? – awkward sentence”

• Page 10, line 218-219, “This could be due to the Ethiopian health system suffers from a lack of adequate medical care and human resources to meet more” There are grammatical errors in this sentence, and it seems like shortage or inadequate supply would be more accurate than “lack” which means there are none.

• Page 12, line 252, “women who were not in union” this is not a clear way to sayunmarried.

• Page 12, line 254 “This might be due to married women had spousal support in making health care decisions towards maternal health service utilization as well as economic and social support” grammatical errors.

• Page 14, line 297, it is better not to say “can’t” and “didn’t” – can not and did not

• Page 14, line 303, “showed that there is the health facility delivery utilization by the reproductive age women has been significantly varied across countries in East Africa.” Sente

**Authors’ response**: Thank you reviewer for your constructive comments. We extensively modified the document by taking into your comments. (See the revised manuscript)
