# Supplementary material for: Pooled prevalence and associated factors of health facility delivery in East Africa: Mixed-effect logistic regression analysis
Source: PLoS One. 2021 Apr 23;16(4):e0250447. doi: 10.1371/journal.pone.0250447 (PMC8064605; doi:10.1371/journal.pone.0250447)
Supplement: Attachment — Submitted filename: Point by point response.docx [file pone.0250447.s003.docx]

**Point by point response for editors/reviewers comments**

**PLOS ONE Journal**

**Manuscript title:** Pooled prevalence and associated factors of health facility delivery in East Africa: A pooled analysis of Demographic and Health Surveys/ Pooled prevalence and associated factors of health facility delivery in East Africa: Mixed-effect logistic regression analysis

**Manuscript ID:**  PONE-D-20-19261R1

**Dear editor/reviewer.**

Dear all,

We would like to thank you for these constructive, building, and improvable comments on this manuscript that would improve the substance and content of the manuscript. We considered each comment and clarification questions of editors and reviewers on the manuscript thoroughly. Our point-by-point responses for each comment and question are described in detail on the following pages. Further, the details of changes were shown by track changes in the supplementary document attached.

**Response to Editors’ comment**

1. The following concerns were not adequately addressed by the author:

The authors addressed some of the points raised by the reviewers adequately. However, some key points and concerns were not addressed in the revised draft.

**Authors’ response**: Thank you for the comments, we have addressed all the comments. (See the revised manuscript)

2. What did the authors do to make sure that the time dimension is not influencing the results?

The authors stated that the DHS dataset years spanned from 2008 to 2018. This is a long period and they do not provide convincing evidence that year of study did not matter. The approach they adopted of picking a random year 2015 and testing it whether there is a significant difference before or after is not sufficient. Have you considered other approaches, including year fixed effects (dummies) or any other appropriate approach?

**Authors’ response:** Thank you for the comments. We run the model considering the year of the survey as a fixed effect (dummies) considering 2008 as a reference but it was not eligible for the final analysis as it has a p-value < 0.2.

We generate a variable year of the survey as, 2008 (Madagascar), 2011 (Mozambique), 2012 (Malawi), 2014 (Rwanda and Kenya), 2015 (Malawi, Tanzania, and Zimbabwe), 2016 (Burundi, Uganda, and Ethiopia), and 2018 (Zambia). Descriptively the prevalence over time was ranging from 44.5% to 72.5% showed a change over time but the regression was not significant when we considered it as a dummy variable and adjusted with the presence of other predictors. If there is a need to report in the manuscript, we are ready to incorporate it.

3. What is the justification for adopting this design of a pooled analysis?

The statistical justification for adopting the pooled design is not sufficient. The DHS datasets are already large enough. The justification should include meaningful reasons, such as understanding factors common or that apply across the region. The authors should be able to answer why it is a good idea to do this work and the answer cannot just be to increase statistical power.

**Authors’ response**: Thank you for the comments. We provide additional justification considering the direction you provide us. Previously we justified it with respect to the statistical significance of pooled analysis but now based on your critical suggestion we justify the clinical implications of a pooled analysis. (See the revised manuscript)

4. Language use in the manuscript is still inadequate. Here are examples from the abstract:

Page 2, line 18 – “but still home delivery is common in” – This is an informal way to say this.

Page 3, line 53 - “Was far below to achieve” – Sentence structure issue

The entire conclusions section is not well written. There are many awkward sentences such as: “Moreover, special attention should be needed for poor”

This manuscript needs to edited by a professional editor. It does not matter if the editor is a native english speaker or not. It needs professional editing by a professional.

**Authors’ response**: Thank you for the comments. We extensively modified the entire document with the help of language experts at the university. (See the revised manuscript)
