# Supplementary material for: Pooled prevalence and associated factors of health facility delivery in East Africa: Mixed-effect logistic regression analysis
Source: PLoS One. 2021 Apr 23;16(4):e0250447. doi: 10.1371/journal.pone.0250447 (PMC8064605; doi:10.1371/journal.pone.0250447)
Supplement: Attachment — Submitted filename: Point by point response.docx [file pone.0250447.s004.docx]

**Point by point response for editors/reviewers comments**

**PLOS ONE Journal**

**Manuscript title:** Pooled prevalence and associated factors of health facility delivery in East Africa: Mixed-effect logistic regression analysis

**Manuscript ID:**  PONE-D-20-19261R2

**Dear editor.**

Dear all,

We would like to thank you for these constructive, building, and improvable comments on this manuscript that would improve the substance and content of the manuscript. We considered each comment and clarification questions of editors and reviewers on the manuscript thoroughly. Our point-by-point responses for each comment and question are described in detail on the following pages. Further, the details of changes were shown by track changes in the supplementary document attached.

**Response to Editors’ comment**

1. - There are still language errors in the document. Even the first few lines of the abstract have several errors and I am not sure what pocket studies mean?:

"Despite health facility delivery is identified as a key strategy for reducing

maternal and neonatal mortality, it less utilized in many African countries. There are

pocket studies on the prevalence and associated factors of health facility delivery in

different East African countries but the prevalence and significant factors were varied

from study to study." "So, pooled analysis using the nationally representative DHS data of East African 114 countries is vital for understanding associated factors common across countries." Is an informal way to express this idea.

**Authors’ response**: Thank you for the comments. We extensively modified the sentence structure and for any typographical error with the help of language experts at the university. (See the revised manuscript)

2. The discussion about how the time dimension was handled also need to be included in the manuscript.

**Authors’ response**: Thank you for the comments. We included in the method section. (See the revised manuscript)
