# Supplementary material for: Pooled prevalence and associated factors of health facility delivery in East Africa: Mixed-effect logistic regression analysis
Source: PLoS One. 2021 Apr 23;16(4):e0250447. doi: 10.1371/journal.pone.0250447 (PMC8064605; doi:10.1371/journal.pone.0250447)
Supplement: Attachment — Submitted filename: Point by point response.docx [file pone.0250447.s005.docx]

**Point by point response for editors/reviewers comments**

**PLOS ONE Journal**

**Manuscript title:** Pooled prevalence and associated factors of health facility delivery in East Africa: Mixed-effect logistic regression analysis

**Manuscript ID:**  PONE-D-20-19261R3

**Dear editor.**

Dear all,

We would like to thank you for these constructive, building, and improvable comments on this manuscript that would improve the substance and content of the manuscript. We considered each comment and clarification questions of editors on the manuscript thoroughly. Our point-by-point responses for each comment and question are described in detail on the following pages. Further, the details of changes were shown by track changes in the supplementary document attached.

**Response to Editors’ comment**

1. There are still language errors even in the abstract:

Examples:

1- "This study showed that health facility delivery in East African countries is low." It should say the percentage or rate of health facility delivery)

2- "These findings suggested that maternal and child health programs should enhance health facility delivery in rural residents and poor households by enhancing maternal education, and ANC service utilization." This sentence is poorly written and hard to understand.

Please have the manuscript professionally edited. There are still errors even in the abstract.

**Authors’ response**: Thank you Editor for the comments. We extensively edited the whole manuscript for any typographical and grammatical errors. Besides, it is reviewed by language experts at the university. (See the revised manuscript)
