# Supplementary material for: Pooled prevalence and associated factors of health facility delivery in East Africa: Mixed-effect logistic regression analysis
Source: PLoS One. 2021 Apr 23;16(4):e0250447. doi: 10.1371/journal.pone.0250447 (PMC8064605; doi:10.1371/journal.pone.0250447)
Supplement: Attachment — Submitted filename: Point by point response.docx [file pone.0250447.s006.docx]

**PLOS ONE**

**Point by point response for editors comments**

**Manuscript title:** Pooled prevalence and associated factors of health facility delivery in East Africa: Mixed-effect logistic regression analysis

**Manuscript ID: PONE-D-20-19261R4**

**Dear editor/reviewer.**

Dear all,

We would like to thank you for these constructive, building, and improvable comments on this manuscript that would improve the substance and content of the manuscript. We considered each comment on the manuscript thoroughly. Our point-by-point responses for each comment and question are described in detail on the following pages.

**Response to Editors comment**

1. Journal Requirements:

Please review your reference list to ensure that it is complete and correct. If you have cited papers that have been retracted, please include the rationale for doing so in the manuscript text, or remove these references and replace them with relevant current references. Any changes to the reference list should be mentioned in the rebuttal letter that accompanies your revised manuscript. If you need to cite a retracted article, indicate the article’s retracted status in the References list and also include a citation and full reference for the retraction notice.

Authors’ response: Thank you for the concerns. We assessed all the references cited in the manuscript and there is no retracted refrences.

2. The language of the manuscript is still inadequate. The manuscript cannot be accepted in its current form.

There are still language errors - even in the abstract.

This is the conclusions section in the abstract of the paper:

Conclusion: This study showed that the proportion of health facility delivery in East African

countries is low.

*Clear

'Country, residence, maternal education status, husband education status,

marital status, occupation status, wealth status, ANC visit, health care access, parity, type of

gestation and wanted pregnancy were significantly associated factors of health facility delivery.'

*This sentence is typically reported as part of the results and not conclusions section

Enhancing maternal education and ANC visit would increase delivery at a health facility. We

recommended maternal health programs targeting improving health facility delivery should emphasize for rural residents.

* You cannot say enhance ANC visit? what does this mean? Also would increase delivery at a health facility implies that you established causality, which is not the case so you should say likely to increase or expected to increase instead.

* the sentence "We recommended maternal health programs targeting improving health facility delivery should emphasize for rural residents." is grammatically incorrect and poorly structured

**Authors’ response**: Thank you for the commnets. We extensively edited and corrected the sentence structure with the help of language experts at the university. (See the revised manuscript)
